# Positioning of term infants during delivery room routine handling – analysis of videos

**DOI:** 10.1186/1471-2431-14-33

**Published:** 2014-02-04

**Authors:** Dimitrios Konstantelos, Heidrun Gurth, Renate Bergert, Sascha Ifflaender, Mario Rüdiger

**Affiliations:** 1 Department of Neonatology and Pediatric Intensive Care, Medizinische Fakultät Carl Gustav Carus, TU Dresden, Fetscherstraße 74, Dresden 01307, Germany

**Keywords:** Newborn, Positioning, Delivery room, Video recording, Agitation

## Abstract

**Background:**

Delivery room management (DR) of the newly born infant should be performed according to international guidelines, but no recommendations are available for an infant’s position immediately after birth. The present study was performed to answer the following questions: 1. How often is DR-management performed in term infants in side position? 2. Is routine DR-management possible in side position? 3. Is there any benefit of side position with respect to agitation or vital parameters?

**Methods:**

Cross-sectional study of video-recorded DR-management in term newborns delivered by C-section in 2012. Videos were analysed for infant’s position, administered interventions, vital parameters and agitation.

**Results:**

187 videos were analysed. The Main Position (defined as position spent more than 70% of the time) was “supine” in 91, “side” in 63 and “not determinable” in 33 infants. “Supine” infants received significantly (p < 0.001) more often stimulation (12.5% of the total time) than “side” infants (3.9% of time). There were no differences between both groups with regard to suctioning; CPAP was exclusively (98%) administered in supine position. Newborns on side were less agitated than those on supine. There was a trend towards a better oxygenation in “side” positioned infants (p = 0.055) and significantly (p = 0.04) higher saturation values in “left-sided” infants than “right-sided” infants at 8th minute. “Side” positioned infants reached oxygen saturation values >90% earlier than “supine” positioned infants (p = 0.16).

**Conclusions:**

DR-management is feasible in the side position in term infants. Side position seems to be associated with reduced agitation and improved oxygenation. However, it remains unclear whether this represents a causal relationship or an association. The study supports the need for a randomized controlled trial.

## Background

Routine handling of the newborn immediately after birth as well as resuscitative interventions in the delivery room (DR) should adhere to international guidelines, which are based on scientific evidence [1-3]. Recommendations describe different aspects of infant handling in great detail, but only limited information regarding positioning of the newborn can be found. Whereas the guideline in 2000 suggested placing the newly born infant supine or lying on its side [4], these suggestions were deleted in subsequent guidelines [1,5].

In daily routine, infants are placed on their back upon their arrival on the resuscitation table in most hospitals. However, side (“fetal”) positioning is becoming more popular in some German hospitals because it is argued that infants feel more comfortable and are less agitated. Side position, however, could have a negative effect on postnatal respiratory adaptation with subsequently increased need for respiratory support since the upper lung will be aerated first [6]. Prior to a change in current practice of infant’s positioning during DR-management, data concerning efficiency and side effects are required. O’Donnell and co-workers are running a study to compare if preterm infants breathe more effectively after birth when placed on their left side than placed on their backs [7]; however up until now there is a lack of data.

In the present study, data extracted from routine video-recordings of DR-management in term infants were used to answer the following questions: 1. How often are term infants cared for in side position? 2. Can medical interventions be efficiently administered in side-positioned term infants? 3. Is there evidence for a benefit of side position with respect to agitation or vital parameters (heart rate and oxygen saturation)? The data of the present study suggests an alternative hypothesis that can be assessed by a randomized controlled trial on positioning of term infants during DR-interventions.

## Methods

The study was conducted at University hospital of Dresden, a level 3 perinatal center with more than 2000 births and about 120 very low birth weight infants per year, between January and December 2012. DR-management was recorded for all newborns born by planned or emergency caesarean sections during the morning shift.

All medical interventions were performed according to the decision of the attending pediatrician and in adherence to local recommendations. There were no general recommendations regarding the positioning or type of stimulation of the infant. Newborns that were put in a lateral position were placed on either right or left side, according to the preferences of the attending physician.

### Ethics

In our institution video recording is part of the routine patient care and approved by the local ethic committee (Ethikkomission an der Technischen Universität Dresden), therefore all data can be used for research purposes as long as no patient or medical care worker can be identified [8]. Videos were stored for later analysis in a way that no identification of individual patients was possible. Recording did not affect care of the individual patient.

### Video recording and analysis

Video-recording was performed by two web-cameras that were connected to a notebook out of the hospital network. One camera was attached on the upper left side of the radiant warmer and one in front of the pulse oximeter monitor. Signals of both were combined by using special software (Manycam virtual webcam – version 3.0, Manycam©) in order to create a picture in picture icon. The recorded picture featured a complete view of the medical interventions with a small picture quadrat on the lower right corner showing the vital parameters. The section of the recorded image was chosen in a way to be unable to identify the caretaker. Recording started with the arrival of the infant on the resuscitation table.

In the present study, videos were analysed from term born babies delivered by c-section, because in our department only c-sections are routinely attended from a paediatrician. If postnatal adaptation was complicated and resuscitation became necessary (e.g. intubation, cardiac massage or drugs) videos were excluded, since the aim of the study was to analyse effect of positioning during “routine care”.

Video analysis was performed through Interact® software (Interact® - Mangold International GmbH), a video based observation software with the ability of qualitative and quantitative data analysis. The inter-rater variability among 7 raters showed a divergence of 1.77 seconds for the duration of an intervention and 1.8 seconds for the time point an intervention occurred. Since many different interventions occur during the first minute at the resuscitation table, analysis in the present study was started after the first minute. Analysis was stopped 10 minutes later or at the time the infant was finally removed from the resuscitation table (whichever came first).

### Definition of parameters

#### Position of the infant

The position of the infant was characterized as either “supine”, “right” or “left” for each time point of analysis. The time the infant was absent from the resuscitation table was excluded. After summing-up of all time intervals, total and relative duration in each position was calculated. Furthermore, for each infant a “Main Position” was determined, defined as the position in which a newborn spent more than 70% of the time. We hypothesized a priori that spending 70% of the total time on a particular position would be sufficient in order to analyse the effect of the individual position. According to that definition, Main Position of infants were categorized as either “side” (>70% on either left or right side), “supine” (>70% in supine position) or “not determinable”. For infants in side position the position was further differentiated into “left-sided”, “right-sided” or “left/right-sided” (>70% of the time on either left, right or both sides respectively).

#### Duration of CPAP application

In order to analyse the impact of infant’s positioning on medical interventions, videos were analysed with respect to the total and relative duration of CPAP-administration. CPAP was considered to be administered when either a mask (Neopuff™ Infant T-Piece Resuscitator, Fisher & Paykel Healthcare Limited) was placed over the nose and mouth, or a pharyngeal tube was in place and connected to the ventilator. For the present analysis efficacy of CPAP administration was not evaluated and no supplemental oxygen data were included. However, newborns that received intermittent position pressure inflations were included.

#### Other medical interventions

The absolute and relative time of other medical interventions such as time of stimulation or suctioning (and number of attempts of suctioning) was analysed.

#### Newborn agitation

A simple three-point scoring system was used in order to evaluate newborns agitation. In accordance with this system infants received 1 point if they were lying calm without moving their extremities, grimacing or crying; 2 points if they were significantly grimacing, shaking and/or stretching their extremities and 3 points if they were extremely irritated with excessive crying or moans/groans in addition to movement of extremities. Newborn agitation state was evaluated every minute after the arrival on the resuscitation table. No score was given to newborns with floppy muscle tone and reduced reflexes under CPAP therapy at this particular minute. Agitation score was given by the same person who reviewed the videos. The reviewer had no involvement in the delivery room management of the newborns that have been reviewed. The score has been validated internally by 5 different observers and no inter-rater differences have been found.

#### Vital parameter

Heart rate (HR) and O_2_ saturation (functional SpO_2_) were analysed at the time points of 5 and 8 minutes according to the monitor (Philips Agilent M3, M3046A, ©Koninklijke Philips N.V.) display. The pulse oximeter sensor was placed preductal (on the right hand). Only reliable readings were used for analysis.

### Statistics

Results are presented in median values and range except from agitation score (mean and standard deviation). Man-Whitney U two tailed test was used to test the null hypotheses except from the values of the first SpO2 >90% occurrence for the two main positions (null hypothesis tested with log-rank test).

## Results

All together 264 videos were recorded during the study period, a total of 187 videos met inclusion criteria for the present study and were analysed. 68 videos were excluded due to prematurity, 8 due to the absence of the monitor and 1 because the newborn required intubation.

Median duration of the analysed video-sequence was 9.9 minutes (ranging between 2.9 and 10 minutes). In 38 videos the newborn was temporarily absent from the resuscitation table in a median time of 25 (7 – 403) seconds.

### Positioning

A total time of 1707 minutes were analysed. During that time infants were positioned “supine” for 1042 minutes (61% of total time), “right” for 516 minutes (30% of total time) and “left” for the remaining 149 minutes (9% of total time).

The Main Position during the procedure was “supine” for 91 newborns, “side” for 63 infants and “not determinable” in 33 infants. Infants with the Main Position “side” were positioned either “right-sided” (n = 47), “left-sided” (n = 10) or “right/left-sided” (n = 6).

**Figure 1:**
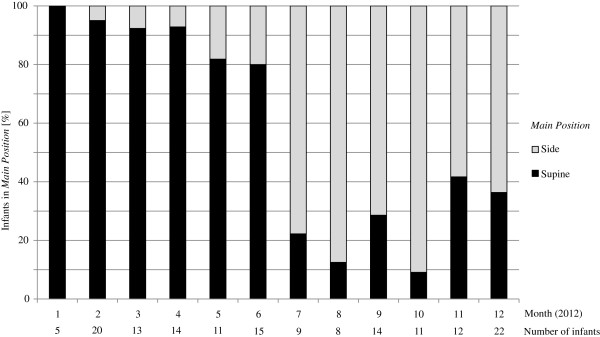
Distribution of the two main positions over time.

The practice of caregivers regarding infant positioning changed over the study period. Whereas more than 80% of infants were managed in supine position during the first half of the study period, less than 40% were in supine position in the second half (Figure 1).

Despite defining a Main Position in the majority of infants, in a large number of infants position was changed frequently. To quantify the frequent change in position, Table 1 shows the number of infants that spent more than one minute in a certain position. Only about 43% of all infants spent all the time exclusively in a single position (supine, left, or right).

**Table 1:** **Number of newborns in *main positions *** **and newborns positioning for more than 1 minute**

***Main position***	**N**	**Supine**	**Right**	**Left**
Supine	91	63*	2	2
Right	47	5	14*	0
Left	10	2	1	3*
Right/left	6	1	5	5
Not determinable	33	11	17	6

*Exclusively in *Main Position.*

### Effect of infant’s main position on routine procedures

The Main Position seemed to have no effect on the frequency of postnatal stimulation. 77 of 91 (85%) newborns in the Main Position “supine” and 45 of 63 (71%) in the Main Position “side” received stimulation. The frequency of stimulation was also not different for different side positions (68% right-sided, 80% left-sided and 83% left/right-sided). Main Position, however, had a significant (p < 0.001) effect on total time spent for stimulation. Whereas “supine” infants were stimulated in median for 12.5% (range 0–62) of the total time, “side” infants were only stimulated for 3.9% (0–80). The relative time of stimulation did not differ between left-sided [5.8% (0–35)] and right-sided infants [3.9% (0–27)].

Oro-pharyngeal suctioning was performed in a total of 40 newborns [median 2 (range 1–6) times per infant]. 28 of 91 (31%) “supine” infants were suctioned for a median of 23 sec (4–87) per infant, 13 of 47 (28%) of “right sided” infants (median 25 sec, range 5–80) and 1 of 10 (10%) of “left sided” infants (2 sec).

43 of the analysed infants received respiratory support with CPAP. The majority (n = 40) were exclusively treated in the supine position. CPAP was started in one infant on the right and in another on the left side; however, both infants were switched to the supine position during CPAP application. Only one infant received CPAP strictly on the right side.

### Effect of infant’s main position on vital parameters

Infant’s Main Position did not affect heart rate. As shown in Table 2, there were no differences between the groups at either 5 or 8 minutes. In infants without measurements, there was either a loss of data or the sensor was not functioning at that particular moment.

**Table 2:** **SpO**_**2 **_**and heart rate values for different *main positions *** **at 5 and 8 minutes**

**Position**	**5 minutes**			**8 minutes**		
	**Number of available readings**	**Heart rate**	**SpO**_**2**_	**Number of available readings**	**Heart rate**	**SpO**_**2**_
Supine	82	157 (60–215)	88 (55–100)	62	157 (124–212)	93 (70–100)
Side	55	163 (112–200)	92 (57–100)^a^	52	163 (106–245)	94 (80–100)
Right sided	41	163 (112–200)	92 (78–100)	39	162 (106–204)	93 (80–100)
Left sided	9	164 (133–200)	94 (80–100)	7	177 (129–245)	97 (94–100)^b^
Right/left sided	5	162 (145–176)	92 (57–95)	6	165 (132–171)	95 (83–98)

Shown are median (range) of available valid readings. ^a^p = 0.055 vs. Supine, ^b^p = 0.04 vs. Right sided.

**Figure 2:**
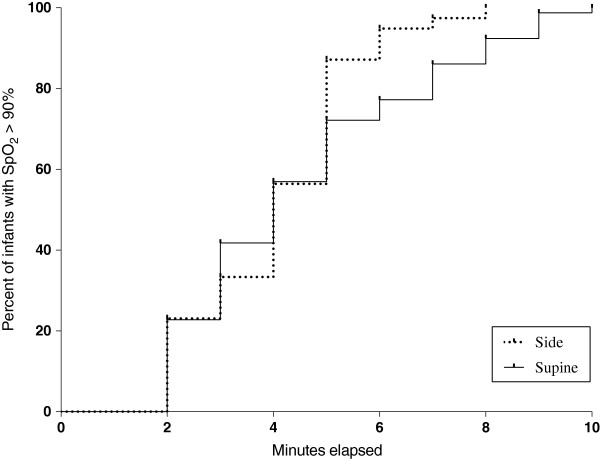
First occurrence of SpO2 over 90% for the two main positions.

Infants with Main Position “side” achieved oxygen saturations above 90% earlier than “supine” infants, the difference however was not statistically significant (p = 0.16) (Figure 2). With regard to absolute values, oxygen saturation tended (p = 0.055) to be lower in “supine” infants at 5 minutes and no differences were found between the two groups at 8 minutes. Comparison of saturation in different side positions revealed no differences at 5 minutes; however there were significantly (p = 0.04) higher values in “left-sided” infants at 8 minutes when compared to right sided infants.

### Effect of infant’s position on agitation

At every time point agitation score was significantly (p < 0.01) lower in infants positioned “side” when compared to “supine” infants (Table 3). There were no differences in the agitation score with regard to position on either left or right side.

**Table 3:** **Agitation score for the actual position over time**^**a**^

**Minute**		**Actual position**			
	**Supine**	**n**	**Side**	**n**	**p**
2	2.17 (0.7)	99	1.83 (0.76)	75	0.005
3	2.12 (0.72)	93	1.9 (0.8)	82	0.07
4	2.01 (0.71)	86	1.73 (0.8)	82	0.02
5	1.95 (0.77)	86	1.71 (0.72)	81	0.05
6	1.92 (0.77)	86	1.57 (0.76)	80	0.005
7	1.96 (0.79)	85	1.59 (0.74)	73	0.004
8	2.03 (0.65)	77	1.6 (0.69)	65	<0.001
9	1.9 (0.71)	77	1.55 (0.64)	56	0.007
10	2.14 (0.68)	66	1.62 (0.7)	45	<0.001

^a^Data are displayed as mean (standard deviation).

## Discussion

No recommendations regarding position of the infant during medical support of postnatal adaptation are given in international guidelines, most likely due to a lack of sufficient scientific data. The present study provides for the first time data on DR-management showing: 1. A substantial number of term infants is already cared for in side position, a habit that became more popular over the one year study period. 2. Routine interventions can be administered in side-positioned term infants. 3. Infants in side position were less agitated and seemed to have better saturation values. Whereas the present data represent– to our knowledge – the first scientific evidence that DR-management in side position is feasible and potentially beneficial, the study rises questions that can be answered only by subsequent controlled clinical trials.

In our institution the preferred position of placing infants during the postnatal adaptation period was “supine”, followed by right and left side. Although a frequent switch from one to another position was noticed, there was no detectable pattern to explain choice of position, except for caretaker’s individual preferences. Furthermore, we noticed a switch in the preferred position from “supine” to “side” during the study period. Since video recording is a standard procedure in our unit and a structured feed-back should be performed to improve quality of care, it cannot be excluded that a certain habit of individual caregivers is “contagious”. Just by watching the treatment provided by other caregivers, the individual habit can change [8]. Since no official change of institutional guidelines occurred during the study period, this finding supports the great potential (but also possible harm) of using video monitoring in routine care.

Results of the present study suggest that routine procedures like stimulation and suctioning can be performed without any problems in either position. However, it is noteworthy that “supine” infants received stimulation for a longer time than infants in “side” position. The reason for the extended stimulation periods in supine position remains speculative. Stimulation that was performed in the first minute in the context of drying and towel change was excluded from analysis. Thereafter, infants included in the study were primarily stimulated to improve respiratory efforts. Infants in supine position (that received more stimulation) were more agitated; however, vital parameters were not better in these infants (there was even a tendency toward lower saturation values). Since stimulation did not have a beneficial effect and caused more agitation, extended stimulation of vigorous term newborns seems to be questionable [9,10]. Although there are studies in premature infants showing that crying is an unnecessary process leading to an increased heart rate, blood pressure and desaturations [11,12], little is known about whether increased agitation causes harm in term newborns immediately after birth. Despite the long history of using stimulation during infant’s resuscitation and its frequent use in routine DR-management [13], limited scientific data concerning benefit or harm of that intervention warrant further research.

Respiratory support was almost exclusively administered with the infant in supine position. In 2 infants CPAP was initially started in side position, but both infants were quickly switched to supine position. A possible explanation for these findings is that caretakers are more familiar with supine position and have a feeling of steadiness that the infant gives while lying with its back on the resuscitation table. According to the experience in other institutions, there will be no problem in administering CPAP in side position. Moreover it is argued that CPAP application with the newborn on side and caretakers hand on newborns back, could even provide more (tactile) information concerning infant’s respiratory efforts. It could be speculated that the reduced agitation in side-placed infants improves efficiency of CPAP application. However, there are no studies that associate infant positioning during CPAP application with a better outcome.

The position of the infant seemed to have no effect on heart rate. With regard to oxygen saturation, infants in side position tended to achieve values above 90% much earlier than “supine” infants. Additionally, side placed infants had higher saturation values at 5th minute than supine placed infants. However, these findings do not necessarily represent a clinical benefit since oxygen saturations were within the physiological range regardless of infant`s position. Interestingly, infants placed on the left side had a significantly higher saturation at 8 minutes when compared to those on the right side. Based on these findings it could be speculated that left side position is associated with faster postnatal lung aeration.

Whereas the results of the present study are of great interest, data do not allow any conclusions concerning a causal relationship. Since our study is not a randomized controlled trial, it cannot be excluded, that infants that “did well” were more likely to be placed on the side, whereas infants that had more difficulties to adapt (and thus required more support) were placed in supine position. However, our study provides sufficient data for a subsequent randomized controlled trial. According to the present data the trial could test the hypothesis that side-positioned infants are less agitated and do have better oxygen saturation values during postnatal adaptation.

Data of the present study are of interest to other topics as well. Whereas several studies were performed to analyse delivery room management of preterm infants, only limited data are available concerning care of term infants, despite the fact that these infants represent the majority of newborns. The present study shows the great potential of video-recording and subsequent analysis for research in DR-management [8].

## Conclusions

The latest guidelines on newborn resuscitation and delivery room management do not recommend a certain position of the infant [2,3]. According to the present data, side position of the infant is feasible, seems to be associated with improved postnatal adaptation of respiratory function and may be more comfortable for the infant (since it reduces infant’s agitation). Therefore, it seems to be safe to place vigorous term newborns laterally during the process of postnatal adaptation; however results of a randomized controlled trial are required to have sound evidence regarding benefits and possible harm.

## Abbreviations

DR: Delivery room; HR: Heart rate; SpO2: O_2_ saturation.

## Competing interests

The authors declare that they have no competing interests.

## Authors’ contributions

DK and MR conceptualized and designed the study, drafted the initial manuscript, carried out initial analyses, contributed to the acquisition of data. HG, RB and SI contributed to the design of the study and for acquisition of data. All authors read and approved the final manuscript.

## Pre-publication history

The pre-publication history for this paper can be accessed here:

http://www.biomedcentral.com/1471-2431/14/33/prepub
